# Designing an Adaptive Underwater Visible Light Communication System †

**DOI:** 10.3390/s25061801

**Published:** 2025-03-14

**Authors:** Sana Rehman, Yue Rong, Peng Chen

**Affiliations:** School of Electrical Engineering, Computing and Mathematical Sciences (EECMS), Curtin University, Kent Street, Bentley, WA 6102, Australia; sana.rehman@student.curtin.edu.au (S.R.); peng.chen@curtin.edu.au (P.C.)

**Keywords:** underwater visible light communication, pulse position modulation, Li-Fi, software-defined radio, power, bit error rate

## Abstract

The Internet of Underwater Things (IoUT) has attracted significant attention from researchers due to the fact that seventy percent of the Earth’s surface is covered by water. Reliable underwater communication is the enabler of IoUT. Different carriers, such as electromagnetic waves, sound, and light, are used to transmit data through the water. Among these, optical waves are considered promising due to their high data rates and relatively good bandwidth efficiency, as water becomes transparent to light in the visible spectrum (400–700 nm). However, limitations such as link range, path loss, and turbulence lead to low power and, consequently, a low signal-to-noise ratio (SNR) at the receiver. In this article, we present the design of a smart transceiver for bidirectional communication. The system adapts the divergence angle of the optical beam from the transmitter based on the power of the signal received. This paper details the real-time data transmission process, where the transmitting station consists of a light fidelity (Li-Fi) transmitter with a 470 nm blue-light-emitting diode (LED) and a software-defined radio (SDR) for underwater optical communication. The receiving station is equipped with a Li-Fi receiver, which includes a photodetector with a wide field of view and an SDR. Furthermore, we use pulse position modulation (PPM), which demonstrates promising results for real-time transmission. A key innovation of this paper is the integration of the Li-Fi system with the SDR, while the system adapts dynamically using a servo motor and an Arduino microcontroller assembly. The experimental results show that this approach not only increases throughput but also enhances the robustness and efficiency of the system.

## 1. Introduction

As water covers nearly two-thirds of the Earth’s surface, the Internet of Underwater Things (IoUT) has attracted much research interest recently. Research in ocean exploration systems has been growing due to climatic changes and resource depletion [1]. The underwater communication system uses media like radio, sound, and optical waves to carry out wireless communication in water. Radio waves are not a good option for such communication since water acts like a conductor. Also, the equipment used is large, expensive, and requires high power, which causes operational and financial difficulties. Acoustic communication is the most widely used underwater technology but has a limited data rate despite the long link range.

**Figure 1:**
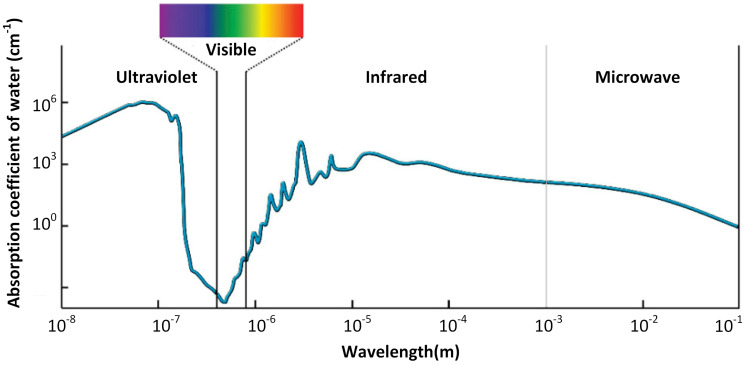
The “transparent window” for light aquatic attenuation [7].

Figure 1 shows the absorption coefficient under the visible spectrum (400–700 nm) underwater. The figure suggests that, considering a suitable wavelength, optical waves within that specific wavelength can be a viable alternative with a reasonable data rate and are probably safe for life underwater. The experiment shows that water becomes transparent to photons under the visible spectrum [2]. In addition, it has the potential to be used in rough conditions and harsh environments as it is easy to deploy [3].

Unlike free-space optical communication, the underwater environment is highly dynamic and influenced by factors such as turbidity, temperature gradients, salinity, absorption, and scattering. Alignment challenges limit underwater optical communication. Underwater visible light communication (UVLC) can be relay-based or software-defined radio (SDR)-based, with light-emitting diode (LED) assembly at the transmitter and photodiodes at the receiver. In this paper, we aim to extend the work presented in our previous study [4]. We have developed a prototype underwater transceiver model to address these limitations, improve alignment, and enhance system performance. The novelties of this design approach are summarized below:

- Designing a UVLC system model by combining Li-Fi (light fidelity) technology with an SDR. Li-Fi transmits the optical signal at high speeds, whereas the SDR conditions the signal using software, thus replacing traditional hardware components such as amplifiers, mixers, and modulators.
- Using pulse position modulation (PPM) instead of conventional OOK (on–off keying), amplitude shift keying, frequency shift keying, and OFDM (orthogonal frequency division multiplexing) for real-time communication.
- The integration of a servo motor and an Arduino microcontroller for the adaptive adjustment of the receiver position concerning the signal direction.

Underwater communication follows the same pattern as free-space optical communication by using the OOK as a primary modulation technique for real-time communication due to its simplicity and high power efficiency. However, since the water channel is complex for light propagation, the UVLC system faces challenges. Research shows [5,6] that PPM and phase shift keying (PSK) are suitable for underwater optical links since they have reasonable data rates and high power efficiency. However, PSK requires higher power and a more complex implementation. PPM and the SDR offer complementary strengths that enhance UVLC systems. PPM ensures power efficiency, while the SDR provides the flexibility to adapt to changing conditions and modulation schemes. By combining these techniques, UVLC systems can achieve improved reliability, adaptability, and efficiency, making them well suited for diverse underwater communication scenarios.

The previous paper [4] measured signal quality at various angular positions by manually adjusting the Li-Fi receiver using PPM and an SDR. In contrast, this paper investigates the effect of automatically adjusting the receiver’s position based on power measurements at different angles using an external setup consisting of Li-Fi, an SDR, an Arduino microcontroller, and a positional servo motor. Specifically, we explore how this automatic adjustment affects the received signal and whether it enables the selection of the optimal direction for signal reception. The received signal strength relative to the receiver angular position is recorded as a power graph versus angular measurements. The findings indicate that the receiver records the maximum power when it is aligned with the direction of the signal. The paper suggests that incorporating an automatic receiver adjustment mechanism based on power measurements at different angles could significantly enhance the performance of underwater communication systems.

This approach could be a key advancement in improving the efficiency of underwater communication systems, particularly in dynamic or moving scenarios where maintaining proper alignment is difficult. The transceiver design proposed in this paper can be easily integrated into wireless sensor networks (WSNs) to support IoUT application through edge computing, as the algorithms can be executed locally.

## 2. Literature Survey

Efforts were made to optimize optical channels by varying system parameters or designing an adaptive system. Tavares et al. in [8] used an angle diversity technique in a receiver to overcome the signal-to-noise ratio (SNR) variation in free-space optical communication. Kaymak et al. in [9] worked on adapting the beam divergence angle in free-space optics for high-speed trains. Despite considerable progress in free-space applications, few visible light communication (VLC) advancements for underwater environments have been made. The challenges of underwater communication, including light attenuation from scattering and absorption, complex optical propagation, and fluctuating environmental conditions, make implementing VLC underwater more difficult.

Simpson et al. in [10] designed a prototype innovative transceiver for multiple users using on–off keying code division multiple access (OOK-CDMA). The proposed receiver offers a wide field of view and can estimate the angle of arrival, with the capability of estimating water quality through optical backscatter. This CDMA research could support the future networking of swarms of uncrewed underwater vehicles. Vali et al. in [11] studied optical Gaussian beam divergence in the lowest-order transverse electromagnetic mode to address the misalignment of the transmitter and receiver. The paper demonstrated that, based on the angle of arrival and the cumulative normalized received power, there is a trade-off between power loss and channel bandwidth. Schirripa et al. in [7] intended to provide an overview of current technologies and potential future developments in underwater optical communication. Sun et al. in [12] reviewed recent advancements in underwater wireless optical communication (UWOC) systems and highlighted the challenge posed by oceanic turbulence, which causes scintillation and misalignment in underwater links. It proposes that using light-scattering-based, non-line-of-sight communication, scintillating-fiber photo receivers, and large photovoltaic cells can reduce the need for complex pointing and tracking systems.

Methods like diffraction beam shaping techniques, relay-based methods, and SDRs were studied to determine the performance of the UWOC channel and how to make it more efficient by making the transmitter or receiver adaptive. Xu et al. in [13] investigated the performance of UWOC in channels with turbulence and misalignment. The diffraction beam shaping method was used to adjust the transmitter distance dynamically. The received power was adjusted through a liquid light crystal valve and a neutral density filter. Salman et al. in [14] proposed a relay-based UWOC system to enhance communication link performance and expand the optical receiver’s reception area. Palitharathna et al. in [15] proposed a dual-hop UOWC system where multiple amplify-and-forward relay nodes support the transmitter–receiver link. The bit error rate (BER) using OOK modulation was analyzed to assess the performance improvements of this relay system. The paper studied the impact of key system parameters, such as transmit power, the number of relays, and the spatial positioning of the attenuation spread, on the system’s overall performance.

Rong et al. in [16] analyzed the factors that affect underwater optical communication systems and compared them with underwater acoustic systems regarding channel capacity, range, and energy efficiency. A multi-hop system was investigated to extend the UWOC range. Chen and Rong, in the paper [17], designed a software-designed UWOC system based on Universal Software Radio Peripheral (USRP) and Python 3.7 capable of transmitting real-time videos through a water tank with a low error rate. Rehman et al. in [4] studied the effect of transceiver alignment on the UVLC system by manually changing the direction of the receiving Li-Fi with respect to the transmitter using an SDR. Power and BER curves for PPM were obtained to study the effect of receiver angular displacement on the received signal. Table 1 on the next page briefly discusses relevant studies in the literature (second column) and summarizes contributions of our work (third column), which addresses gaps in the current research.

## 3. UVLC Channel Modeling

**Figure 2:**
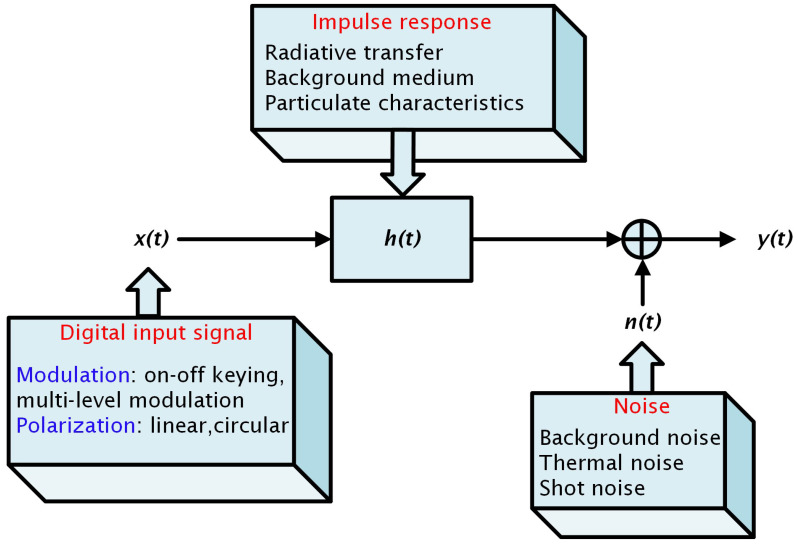
Additive noise channel model [18].

The UVLC channel can be modeled with its impulse response, which describes the response of the channel and dispersion of the received signal in the time domain (also known as temporal dispersion of the received signal). Figure 2 shows the underwater optical channel model.

### System Model

The UVLC system model in Figure 2 can be expressed as

(1) y(t)=h(t)∗x(t)+n(t)

where y(t) is the received signal after passing through a UVLC channel with impulse response h(t), n(t) represents different forms of noise, and x(t) is the modulated transmitted signal.

**Figure 3:**
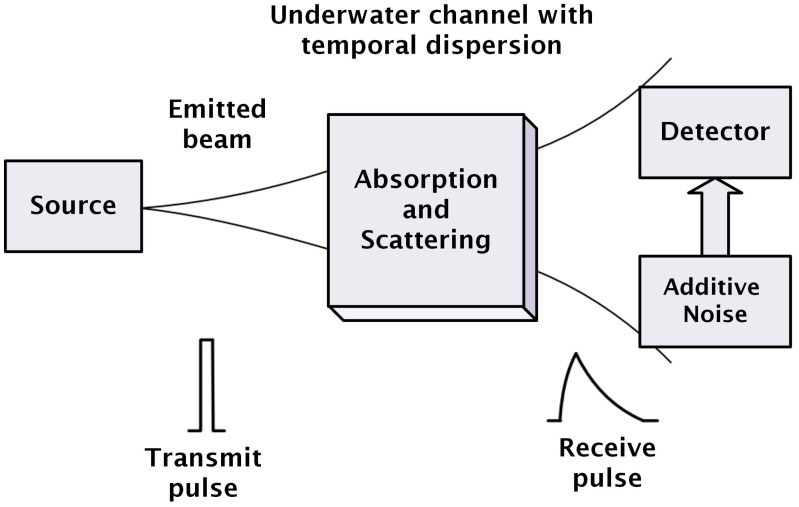
System model and link geometry of the UVLC link [20].

Water is characterized by its inherent optical properties (IOPs) and apparent optical properties (AOPs). The IOPs depend on the aquatic medium whereas the AOPs depend on the water as well as properties of the light field [19]. Absorption and scattering fall under the category of IOPs, whereas irradiance reflectance is an important AOP. Since the latter property mentioned mostly varies with the depth of water, it is not of our concern as, in this project, the depth of water is constant. Here, we only focus on IOPs. Whenever light travels in an aquatic medium, it suffers from absorption and scattering. Absorption is an irreversible process where photons lose energy thermally, whereas, in scattering, they change their direction upon interaction with water molecules and suspended particles [20]. Both are wavelength-dependent. Figure 3 shows the system model for the UVLC link, where the light from the source encounters water molecules and hence suffers from absorption and scattering.

The first step to analyzing any UVLC system is to study its channel characteristics and determine the channel impulse response (CIR). The CIR is used to analyze the effects of channel distortions [2]. Beer’s law is the simplest one, and considers the linear motion of photons. The optical power (*I*) of a photon after a distance (*z*), based on the transmitted power ($I_{o}$), is given by

(2) $$I=I_{o}e^{-cz}$$

where *c* is the extinction coefficient (sum of absorption and scattering coefficients) and *z* is the link range.

The radiative transfer equation (RTE), which considers the linear motion and angular scattering of photons, is given by

(3) $$\left[\frac{1}{v}\frac{\partial }{\partial t}+\vec{n}\cdot \nabla \right]I\left(t,\vec{r},\vec{n}\right)=-cI(t,\vec{r},\vec{n}+\int_{4\pi }\beta \left(\vec{r},\vec{n}{\vec{n}}^{\prime }\right)I\left(t,\vec{r},\vec{n}\right)d{\vec{n}}^{\prime }E\left(t,\vec{r},\vec{n}\right)$$

where *v* is the speed of light, *t* is time,$\vec{n}$ is the direction vector, $\vec{r}$ is the position vector, ∇ is the divergence operator concerning the position vector $\vec{r}$, *I* is the radiance, β is the volume scattering function (VSF), and *E* is the source radiance [21].

The RTE accounts for both the attenuation of light (due to absorption and scattering) and the redistribution of light through scattering processes. It can be solved through the Monte Carlo approach, which works by sending a large number of photons individually through the water body [22], but, despite being accurate and easy to program, it is time-exhausting.

The stochastic model is not only easy to program but also time-efficient. It follows an analytical approach to find the scattering function in order to compute the path loss and the CIR. The position of a photon obtained from this model can be calculated by the probability density function (PDF) but the scattering angle is challenging to deduce from the cumulative distribution function (CDF). Hence, the Henyey–Greenstein (HG) function is used in this model to deduce the scattering phase function, which is critical in finding the path loss and signal quality at the receiving end [23,24,25]. The HG equation is given by [26,27]

(4) $$P_{HG}\left(\theta \right)=\frac{1-g^{2}}{4\pi {\left(1+g^{2}-2gcos\left(\theta \right)\right)}^{3/2}}$$

where $P_{HG}$ is the probability density function of the scattering phase function and *g* is the particle asymmetry factor, which depends on the medium characteristics and is equal to the average of cos(θ). The scattering angle θ can be found by [27]

(5) $$\theta =cos^{-1}(\frac{1}{2g}\left[1+g^{2}-\left(\frac{1-g^{2}}{1+g-2g\xi }{)}^{2}\right]\right)$$

where ξ is a random variable between 0 and 1. From the scattering angle, the power at the receiver can be deduced as [28]

(6) $$P_{r}=\frac{P_{t}e^{-cz}D^{2}cos\left(\phi \right)}{{4z}^{2}{tan}^{2}\left(\theta \right)}$$

where $P_{t}$ is the transmitted power, θ is the scattering angle, *D* is the diameter of the receiver, and ϕ is the inclination angle (angle between the receiver’s normal with respect to the light beam) [28].

The quality of a signal is evaluated based on its SNR, symbolized as *S*. *S* at the receiver is given by

(7) $$S=\frac{P_{r}^{2}}{P_{noise}^{2}}$$

where $P_{noise}$ denotes the noise power. The SNR *S* can be used to find the BER. Different modulation schemes have different BER formulas. The BER for the pulse position modulation (PPM) is given by [29]

(8) $$\eta_{BER}=\frac{1}{2}erfc[\frac{1}{2\sqrt{2}}\sqrt{\frac{SLlog_{2}L}{2}}]$$

where *L* is the PPM level (number of slots needed to encode the message bits) and erfc is the complimentary error function, which is given by [30,31]

(9) $$erfc={\left(2\pi \right)}^{-1/2}\int_{x}^{\infty }e^{-t^{2}}\;dt.$$

We adopt the stochastic model due to its lower computational complexity. In this paper, we study the effect of ϕ in Equation (6) on the system SNR and BER performance. Based on Equations (6) and (7), we can conclude that the alignment between the transmitter (TX) and receiver (RX) plays a crucial role. This alignment is the central focus of our study.

## 4. System Hardware

**Figure 4:**
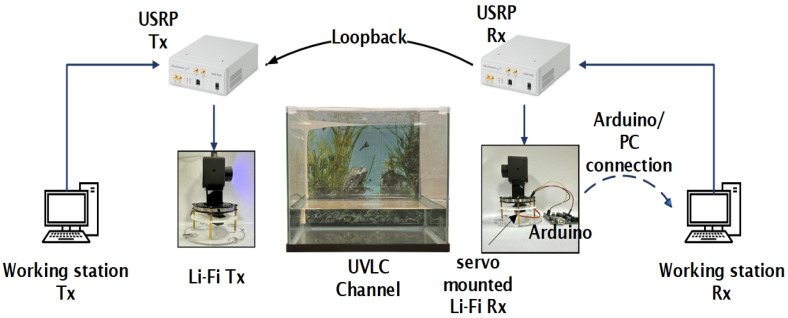
UVLC system model.

The block diagram for the adaptive UVLC system can be seen in Figure 4 below. A modulated 4-PPM signal is created in MATLAB R2023b, which is connected to USRP NI-2920 (manufactured in Austin, TX, USA). The USRP transmits the signal to the Li-Fi transmitter, which converts the electrical signal into light of wavelength 470 nm. The light signal received by the Li-Fi receiver is converted to an electrical signal and sent to MATLAB at the receiving end by the USRP. The two USRPs are connected via loopback. Also, the MATLAB at the receiving end programs the Arduino, which controls the servo motor to change the direction of the Li-Fi Rx to the strongest signal direction for adaptive beam reception.

The Li-Fi R&D kit, which includes both the transmitter and receiver modules from HYPERION Technologie (manufactured in Istanbul, Turkey), was used in the system. This module provides a 20 MHz bandwidth and has a 170-degree detection field of view. A 1-watt blue LED is employed to transmit the signal through water. The analog signal from the USRP is sent to an optical transmitter front-end, which adds a DC bias, converts the electrical signal into blue light, and then transmits the resulting optical signal through the underwater optical channel [17].

## 5. Transmitter Design

**Figure 5:**
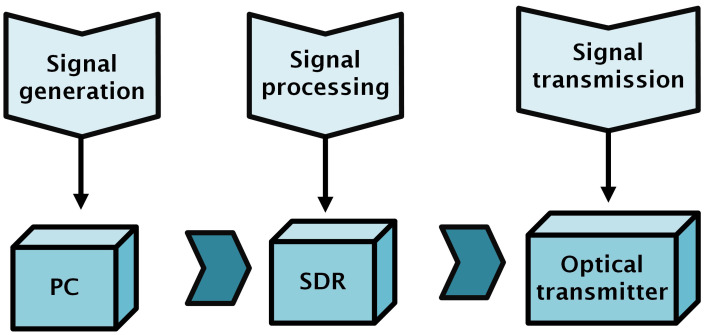
Transmitter design.

A transmitter is responsible for three tasks, i.e., signal generation, processing, and transmission, as shown in Figure 5.

### 5.1. Signal Generation

Signals were generated in MATLAB. A 16-bit pseudo noise (PN) sequence and 4-PPM signal were chosen as the preamble and payload, respectively. The generated signal was modulated using a 2.4 MHz tone signal. While designing the transmitter process in MATLAB, “underrun” was encountered because the writing speed of the host computer was slower than the reading speed of the USRP. Underrun depends on the working station processor speed and the interpolation factor of the radio device. Since the working station had a sufficient processing speed (2.8 GHz), the problem was solved by carefully setting the interpolation factor to 16, corresponding to the tone signal, optical transmitter, and PC. This adjustment allowed for smooth data transmission without underrun.

#### 5.1.1. Modulation Scheme

For creating a payload, the message signal was modulated using pulse position modulation. PPM is a digital modulation technique in which a message signal is encoded in $2^{M}$ possible slots, where *M* is the number of message bits. This modulation scheme has low transmit power and better noise performance [32]. PPM has better noise immunity because it has a constant amplitude at the expense of high bandwidth.

#### 5.1.2. Modulating Signal

Baseband PPM signal transmission can be performed in two ways [4].

(1) Modulating the signal with a carrier whose sampling frequency aligns with the IQ rate of the USRP.

(2) Initializing the carrier frequency of the software-defined radio (SDR) object and transmitting the baseband PPM signal directly.

The second method results in signal demodulation with an unknown offset, which is difficult to calculate. Hence, the first method was chosen [4]. The choice of carrier frequency for the modulating signal is crucial since, if not chosen within the operating range, it can result in affecting the USRP configuration and Li-Fi operation. After several experimental trials, 2.4 MHz was chosen as the carrier frequency. It not only addresses the underrun issue but also helps the optical transceiver to accurately deliver the signal at the receiving end. The interpolation factor of 16, which corresponds to the sampling frequency of 6.25 MHz, satisfies the Nyquist criterion for the tone signal [4].

### 5.2. Signal Processing

A software-defined radio is used to implement digital techniques by replacing traditional hardware like mixers, modulators, and other analog circuits. This makes the SDR more adaptive and flexible to quickly reconfigure in order to handle different signaling techniques. This project uses NI-2920 for software processing. The modulated signal from MATLAB is filtered and upconverted at the daughterboard for pass band transmission [33]. A wide-bandwidth transceiver (WBX) with a frequency range of 50 MHz to 2.2 GHz is the default daughterboard for NI-2920. However, since the desired operating frequency for this specific UVLC system is below 50 MHz, a low-frequency (LF) daughterboard is used instead. The LF daughterboard operates between 0 and 30 MHz. Unlike the WBX, this low-frequency board does not include a local oscillator that could contribute to phase noise.

### 5.3. Signal Transmission

**Figure 6:**
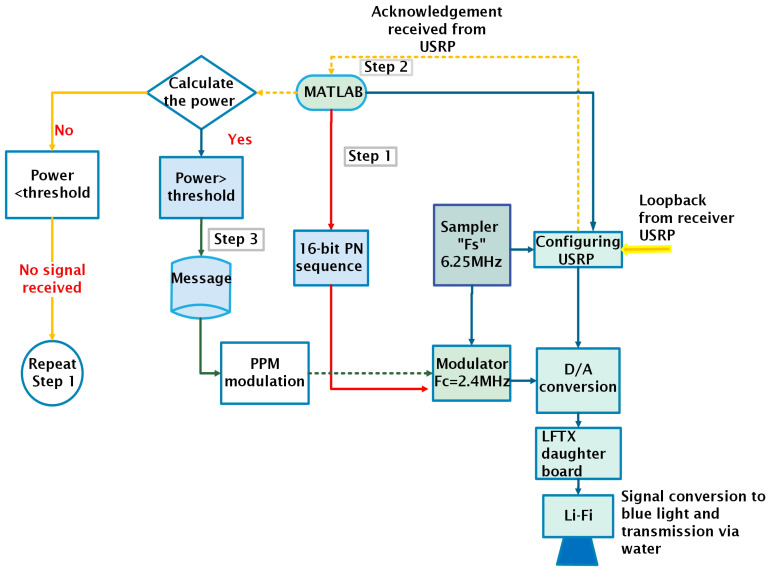
Flow chart for transmitting station.

**Figure 7:**
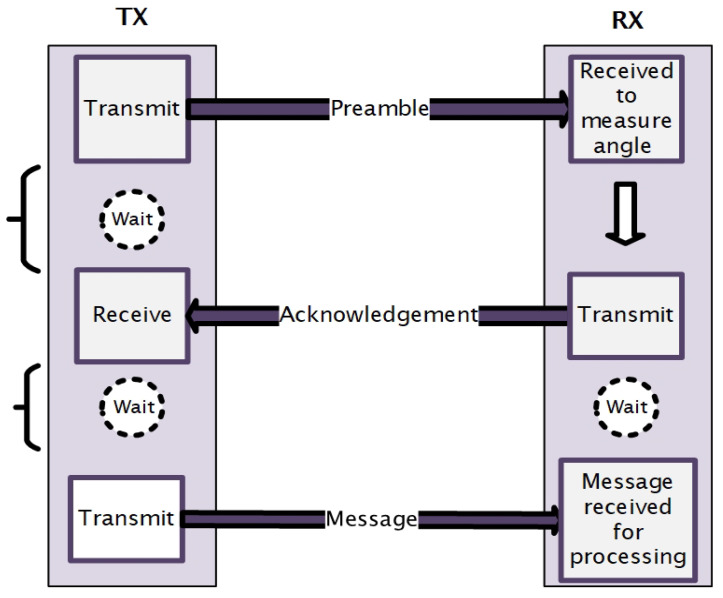
Adaptive transmission.

Figure 6 and Figure 7 illustrate the transmission process in the underwater adaptive visible light communication system.

Figure 6 offers a detailed depiction of each step, with color-coded lines (red, yellow, and green) to highlight the stages of the process, whereas Figure 7 presents a summarized version of the entire transmission process, giving an overview from the preamble transmission to the final message transmission. The detailed descriptions of the process are provided in the following subsections:

Section 5.4: Explains the transmission of the preamble.

Section 5.5: Details the reception of the acknowledgment (ACK).

Section 5.3: Describes the transmission of the message signal.

Together, these figures and sections offer both a visual and written explanation of the full transmission process.

The transmitter follows the following steps.

### 5.4. Transmitting the Preamble

This step is represented by the “red line” in Figure 6. Initially, a 16-bit preamble is created in MATLAB. The signal is modulated with a 2.4 MHz tone signal. The preamble is transmitted via a Li-Fi transmitter.

### 5.5. Receiving the Acknowledgment

This step is represented by the “yellow line” in Figure 6. After receiving the preamble, the receiver calculates and compares the received power at different angles to identify the angle with the strongest signal. It then adjusts its position to align with this optimal angle and generates an acknowledgment (ACK) signal, which is modulated with a 2.4 MHz tone and sent back to the transmitter via loopback. The ACK signal is detected by the LFRx1 of the transmitting USRP, and its power is measured. If the power exceeds a specified threshold, MATLAB prepares the transmitter to send the actual message. If the ACK signal is not received after a certain time, the transmitter starts the process again by transmitting the preamble.

### 5.6. Transmitting the Payload Signal

**Figure 8:**
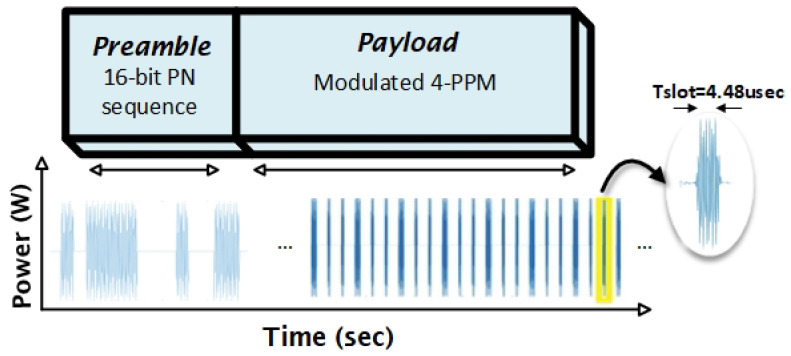
Frame structure of the message signal.

This step is represented by the “green line” in Figure 6. The message signal consists of a preamble and payload, as shown in Figure 8. The payload is a 223.2 KHz, 4-PPM signal modulated with a 2.4 MHz tone signal. The payload along with a modulated 16-bit pseudo-noise (PN) sequence (necessary to calculate the offset frequency at the receiver end) is transmitted via USRP to the Li-Fi transmitter, which is responsible for transmitting the message through water to be received at the receiver.

## 6. Receiver Design

The receiver works in a similar fashion as the transmitter, incorporating the receiving USRP, the optical Li-Fi receiver, and the workstation. Key challenges like overflow and offset frequency are already discussed in [4]. This paper studies the adaptive reception of the signal. Hence, the receiver is modified to deal with this kind of reception by mounting the LFRx to the assembly, consisting of a positional servo motor SG90M (manufactured in China) connected to an Arduino Uno R3 microcontroller (manufactured by Arduino, designed and assembled in Italy), which is controlled by MATLAB.

**Figure 9:**
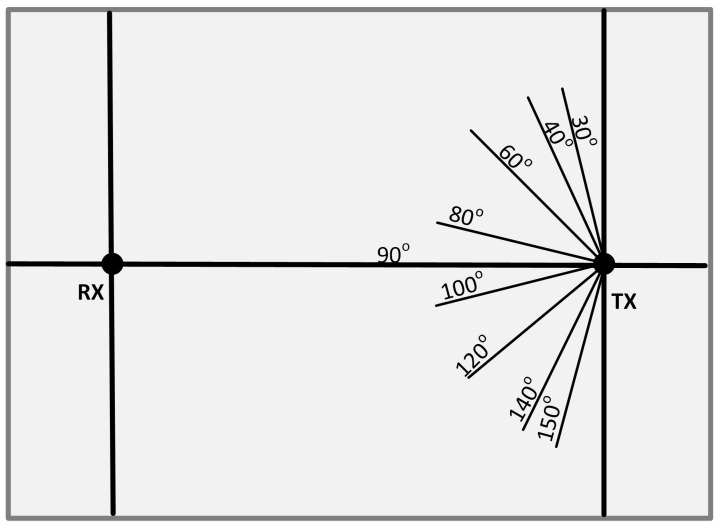
Angular position of the receiver with regard to transmitter.

The angular position adopted by the positional servo motor is calibrated as shown in Figure 9 below. The receiver dynamically adjusts itself between the angles on both sides of the line-of-sight alignment to optimize signal reception. This calibration is different to the manual UVLC system in [4].

### 6.1. Adaptive Reception

**Figure 10:**
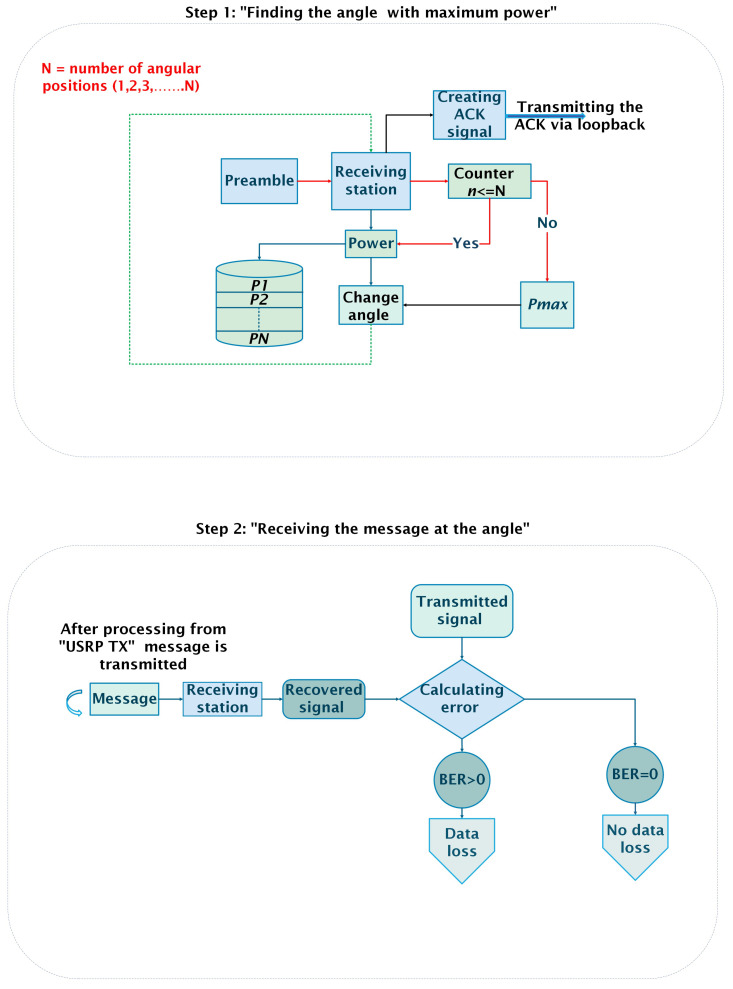
Adaptive receiver design.

**Figure 11:**
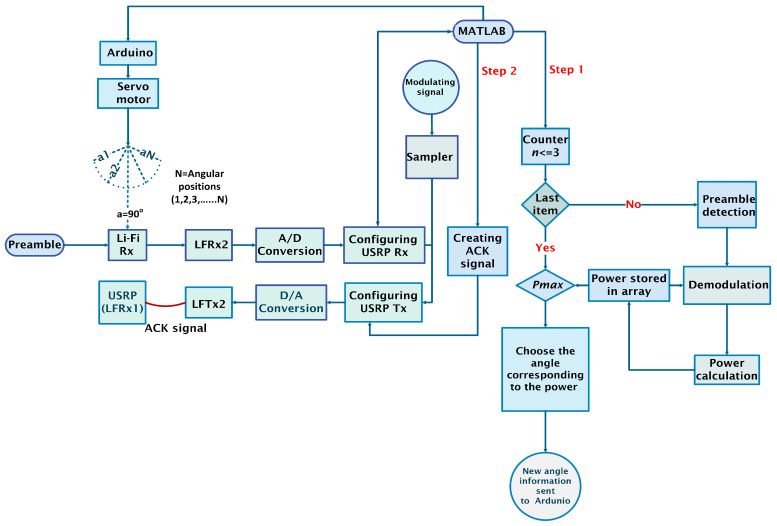
Flow chart for the receiver model to find the angle.

**Figure 12:**
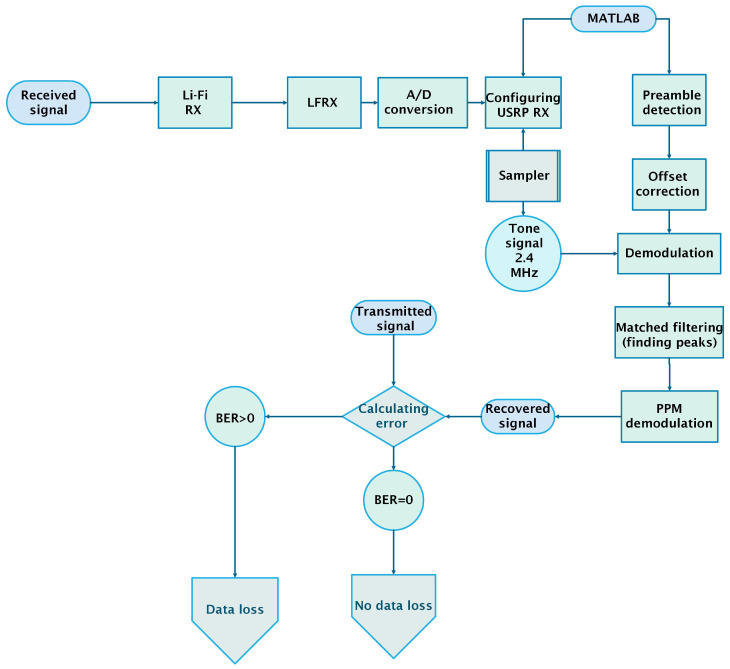
Flow chart for receiving signal [4].

The adaptive receiver design is shown in Figure 10, Figure 11 and Figure 12. The receiver first receives the preamble and calculates the power at three positions, labeled P1, P2, and P3 as shown in Step 1 of Figure 10. These values of power are stored in an array for counter *n*, where n=1,2,3 correspond to angles a1,a2,a3 in Figure 11. Once the counter is greater than 3 (n>3), the receiver compares the power values, adjusts its position to align with the angle that has the maximum power, and then transmits the ACK signal.

The transmitter subsequently compares the power of the received ACK signal with a predefined threshold to determine whether to proceed with sending the actual message. If the message is transmitted, the received signal is recovered, demodulated, and evaluated for the BER as shown in Step 2 of Figure 10. A BER of zero indicates no data loss, while any non-zero BER indicates that some data were lost. Section 6.2, Section 6.3 and Section 6.4 provide a detailed explanation of each step at the receiving end.

### 6.2. Receiving Preamble

Before receiving the actual message, the receiver receives a preamble. Time synchronization is crucial in this process. The program is designed to ensure that MATLAB has enough time to calculate the power at each angle before receiving the next frame for processing. The details of this process can be visualized in Figure 11.

When Li-Fi Rx receives the preamble, it is processed at the USRP and then fed to MATLAB as shown in Step 1 highlighted in Figure 11. Since the comparison is made at three different angular positions, the angular position *N* is 3. For n<=3, the preamble is detected, and power is calculated, which is stored in an array. When n=4, the program exits the loop and the maximum power (Pmax) is selected from P1,P2, and P3 (as shown in Figure 10).The angle information corresponding to Pmax is then sent to the Arduino, which controls the direction of the Li-Fi Rx through the positional servo motor.

### 6.3. Transmitting Acknowledgment

Once the angle with the maximum signal strength is determined and stored, the receiver adjusts its position accordingly. MATLAB then generates the ACK signal, which is modulated and transmitted via loopback to the transmitter. The transmitter, in a waiting mode, awaits the acknowledgment before proceeding with the next steps.

### 6.4. Receiving Payload

After the successful reception of the message signal, the PPM signal is decoded using the matched filter as shown in Figure 12. This filter identifies the peaks, which are then translated into bits. The signal reception process is described in detail in the flow chart presented in [4].

## 7. Experimental Setup

**Figure 13:**
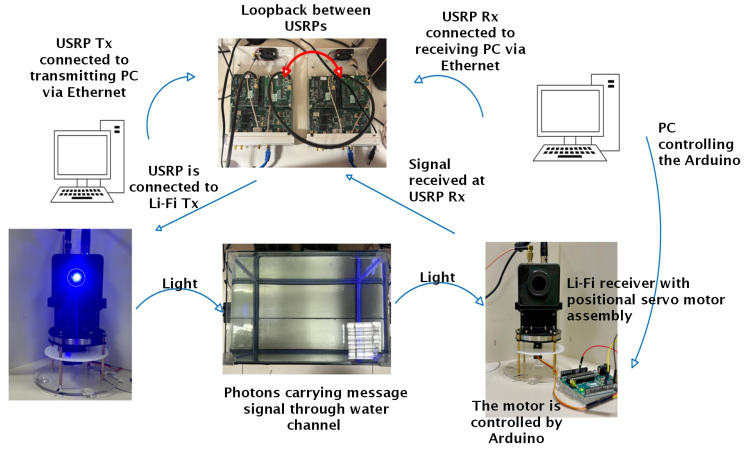
Adaptive UVLC hardware setup.

The real-time hardware setup is illustrated in Figure 13. MATLAB generates a 16-bit PN sequence, which is modulated with a 2.4 MHz tone. This signal is then upsampled and converted into an analog signal using the D/A converter of the USRP. The USRP is equipped with two low-frequency (LF) daughterboards: LFTx1, which is connected to the Li-Fi transmitter, and LFRx1, which is linked to LFTx2 of the receiver USRP. The Li-Fi transmitter is mounted on an assembly, as shown in Figure 13, and the assembly includes a positional servo motor (SG90M) that can be used for non-line-of-sight communication in the future. The Li-Fi system, after adding a DC bias, transmits the signal through a 1-watt blue LED. The experiment was conducted using a water tank (dimensions: 36 cm × 26 cm × 22.5 cm). The tank is covered with a black screen to prevent interference from external light sources. On the receiving end, the Li-Fi receiver is mounted on the same SG90M servo motor. The Li-Fi system removes the DC bias and sends the signal to LFRx2, which downsamples the signal by a factor of 16. The downsampled signal is then digitized by an A/D converter and received by MATLAB.

The system is programmed such that the Li-Fi receiver is initially positioned at 90° (directly facing the transmitting station). The receiver then changes its position to 30°, followed by 40°, 60°, and 80°, respectively. During this time, power is calculated at each position, storing the values in an array for later comparison to determine Pmax. The receiver then shifts back to 90°, calculates the power, and subsequently moves to 150° (with other angles being 140°, 120°, and 100°), where it calculates and stores the power in the array as well.

Meanwhile, the transmitter remains in wait mode. MATLAB at the receiver compares the power at various angles (e.g., 30°, 90°, and 150°), determining that the maximum power occurs at 90°. The receiver then realigns to 90 degrees and generates the ACK signal, which is modulated with the same 2.4 MHz tone. This ACK signal is transmitted back to LFRx1 of the transmitting USRP via LFTx2 of the receiving USRP. The transmitter receives the acknowledgment and calculates the power. If there is any synchronization issue, the program may halt.

The transmitter then compares the power of the received ACK signal with a predefined threshold. If the power is sufficient, the transmitter proceeds to send the preamble and the payload of the 4-PPM, 223.2 kHz signal. The preamble is retransmitted to account for any offset introduced due to differences in the master clock rates (100 MHz) of the two USRPs. The received signal’s frequency is observed to be 2.4046 MHz instead of 2.4 MHz due to this offset (as shown in Figure 12) [4]. After offset compensation, the signal is demodulated and filtered to recover the baseband PPM signal.

PPM demodulation is achieved using a matched filter, which identifies peaks by cross-correlating the PPM signal with a pulse of the same width as the PPM pulse. These peaks are then used to decode the PPM signal. The regenerated signal is compared to the actual transmitted signal to calculate the BER.

## 8. Results

The results are explained in two sections.

Section 8.1 explains the non-adaptive system results, which include the comparison of the 4-PPM and 8-PPM BER and power with respect to the angular position.

Section 8.2 presents the power comparison of the adaptive system with four different sets of angles with 4-PPM. A 4-PPM is chosen over 8-PPM due to better noise performance.

### 8.1. Manual Angular Displacement Results

To make the system adaptive, initial trials were conducted by manually changing the position of the Li-Fi without using any motor. These trials aimed to evaluate the effect of line-of-sight and non-line-of-sight displacement on the system’s performance.

**Figure 14:**
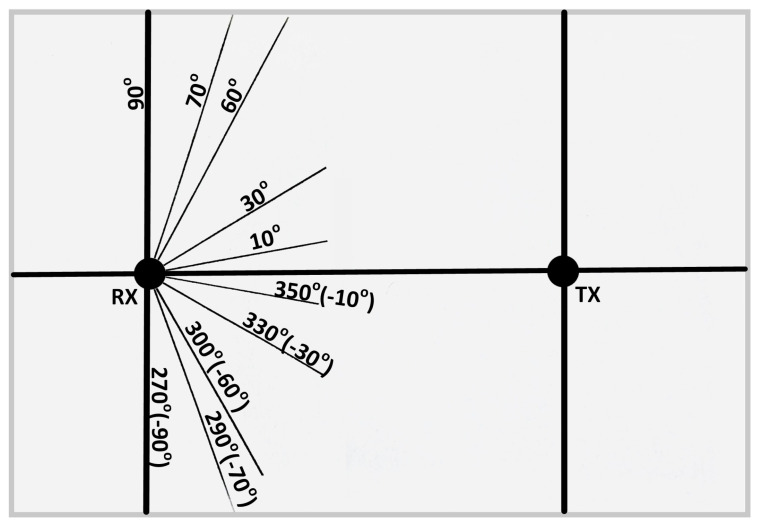
Angular positions of RX and TX for LOS communication [4].

**Figure 15:**
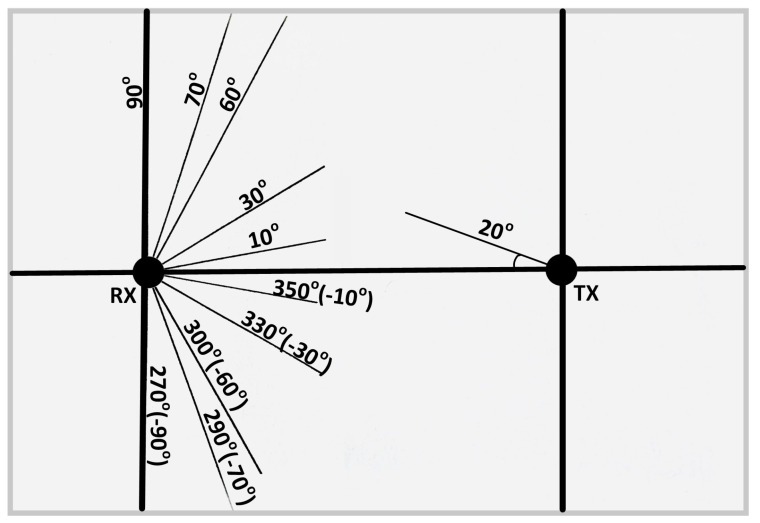
Angular positions of RX and TX for NLOS communication [4].

The reference angular positions of RX with respect to TX are shown in Figure 14 and Figure 15.

**Figure 16:**
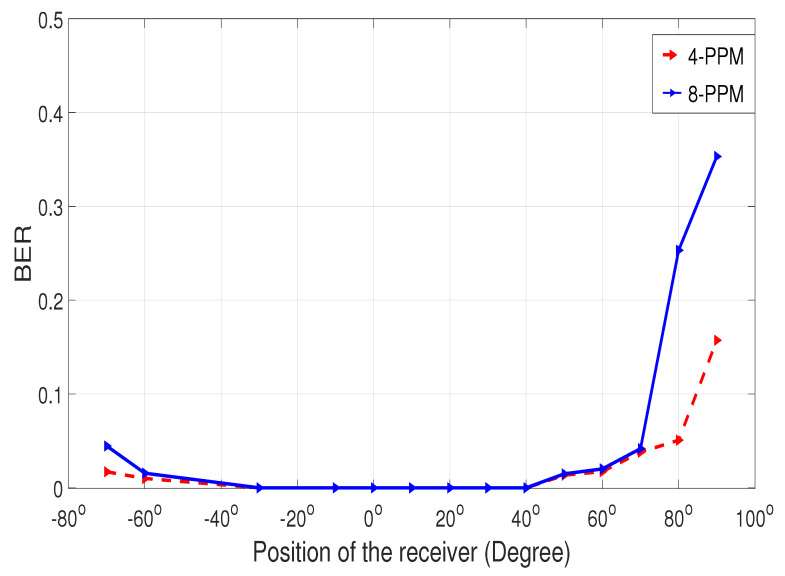
BER analysis for LOS communication.

The experiment for line-of-sight (LOS) communication (angular position shown in Figure 14) suggests that the BER is zero from −30° to 40°, indicating that the transmitter and receiver are properly aligned. However, as the receiver moves to the right or left beyond −30° or 40° as shown in Figure 16, the BER starts to rise, reaching its highest value when the receiver is positioned perpendicular to the transmitter. Additionally, 8-PPM experiences a higher BER at the same angular positions compared to 4-PPM.

**Figure 17:**
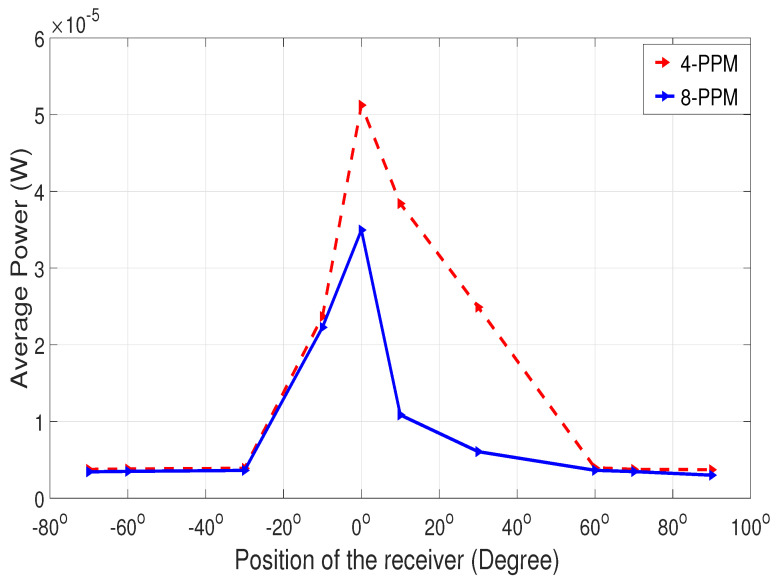
Power analysis for LOS communication.

The power of the signal in Figure 17 is lowest when the receiver is not aligned with the transmitter and reaches its maximum when they are perfectly aligned at 0°. Additionally, the graph indicates that 4-PPM has higher power than 8-PPM because 4-PPM transmits once every four slots while 8-PPM transmits once every eight slots, making 4-PPM more efficient in terms of power.

The experiment for non-line-of-sight (NLOS) communication was conducted in a similar manner to LOS communication. In this case, the transmitter was positioned at a 20° angle, as shown in Figure 15, relative to the LOS distance between the transmitter and the receiver, while the receiver’s position was adjusted as shown in Figure 15.

**Figure 18:**
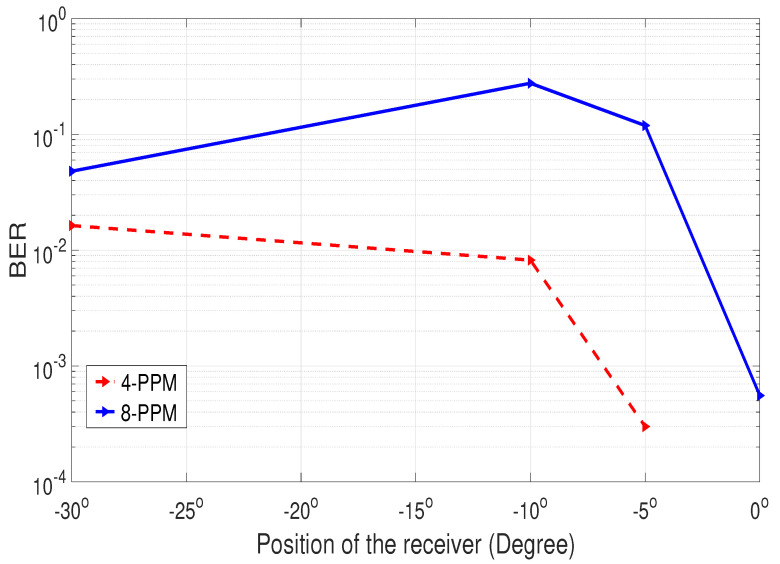
BER analysis for NLOS communication.

Figure 18 demonstrates that the BER increases when the transmitter’s angle of incidence moves out of the receiver’s field of view (FOV). However, once the beam re-enters the receiver’s FOV, the BER begins to decrease.

**Figure 19:**
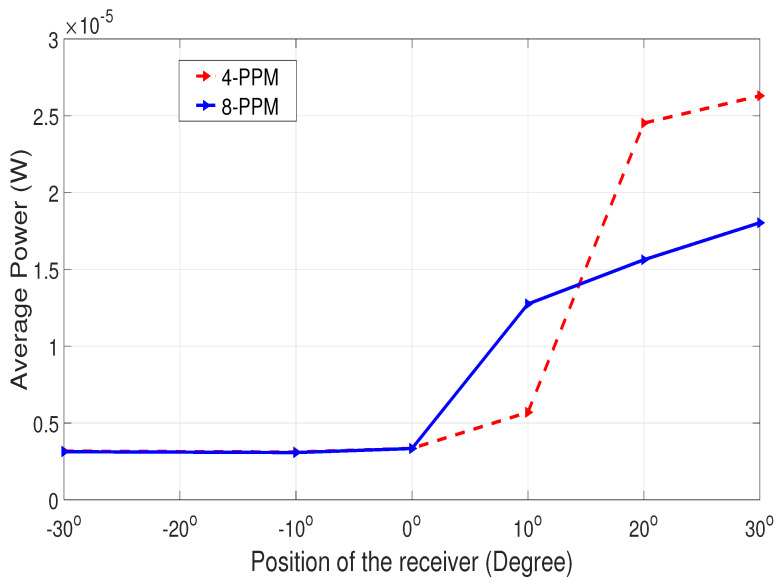
Power analysis for NLOS communication.

In Figure 19, the power for NLOS communication is at its minimum when the incident beam is outside the receiver’s FOV. However, as the transmitter and receiver align, the power increases, leading to improved signal reception.

### 8.2. Adaptive Angular Displacement

After retrieving the results for the BER and power analysis for LOS and NLOS communication, trials on the adaptive alignment system were performed for different sets of angular displacements to study the performance of the adaptive system. The results were challenging to obtain because of the synchronization problem and the repetitive reconfiguration of the USRPs while they were switching between transmitter and receiver mode consistently.

**Figure 20:**
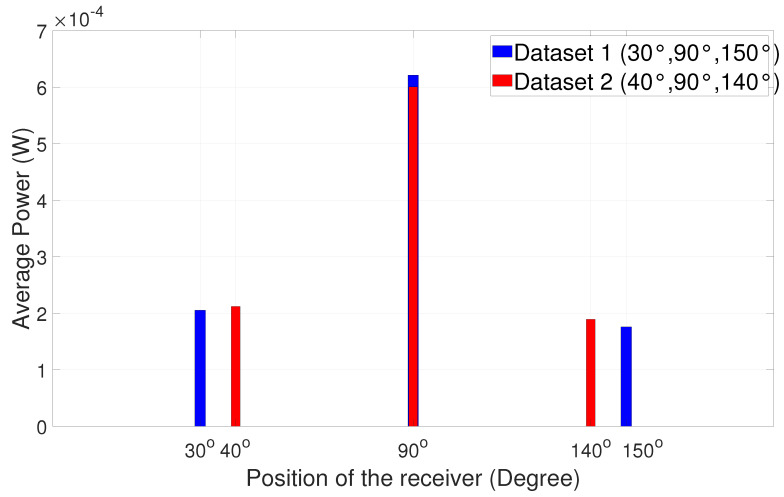
Angular positions vs. power chart 1.

**Figure 21:**
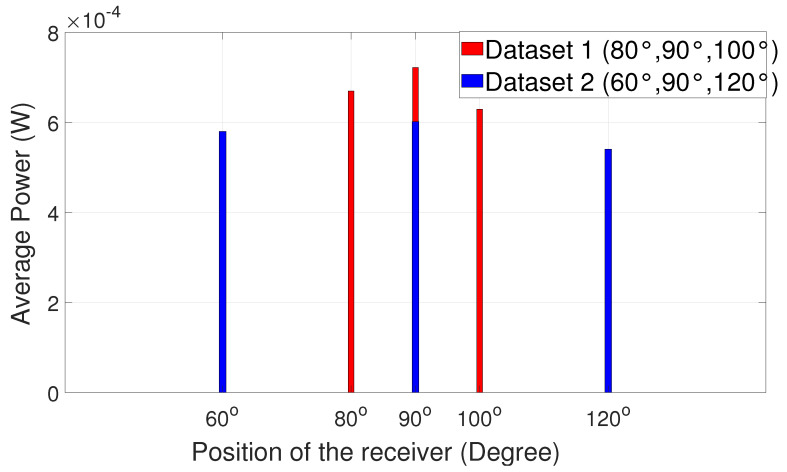
Angular positions vs. power chart 2.

The power analysis comparison for the four sets of angular measurements can be seen in Figure 20 and Figure 21 below. The results of the power analysis are presented in the form of bar charts.

**Figure 22:**
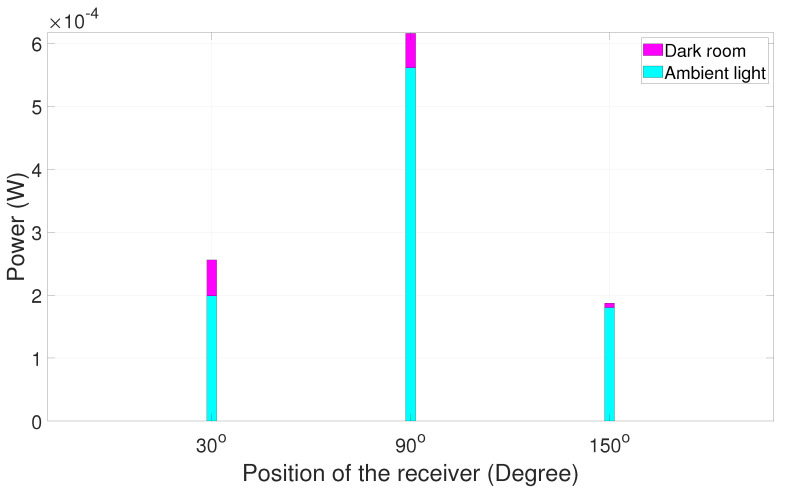
Dark room vs. ambient light power chart trial 1.

**Figure 23:**
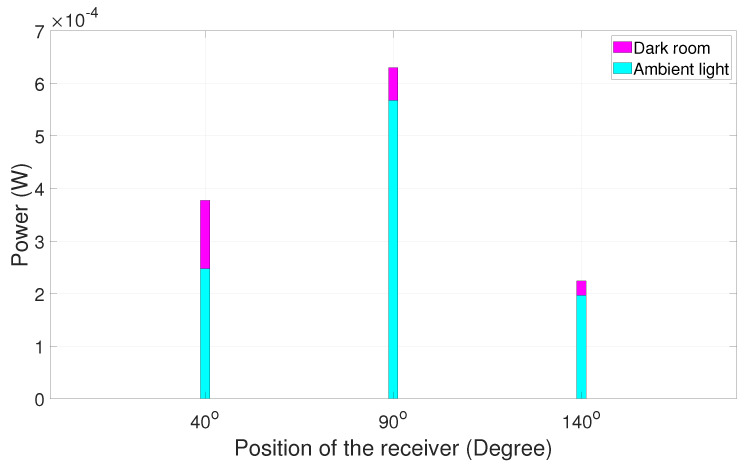
Dark room vs. ambient light power chart trial 2.

**Figure 24:**
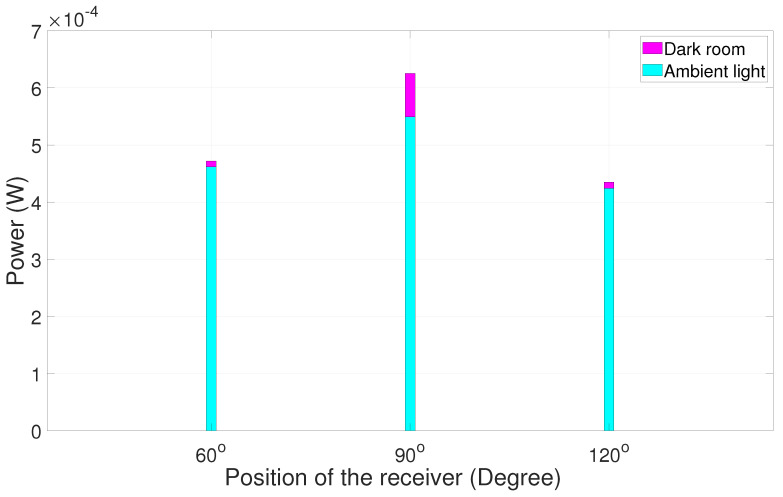
Dark room vs. ambient light power chart trial 3.

**Figure 25:**
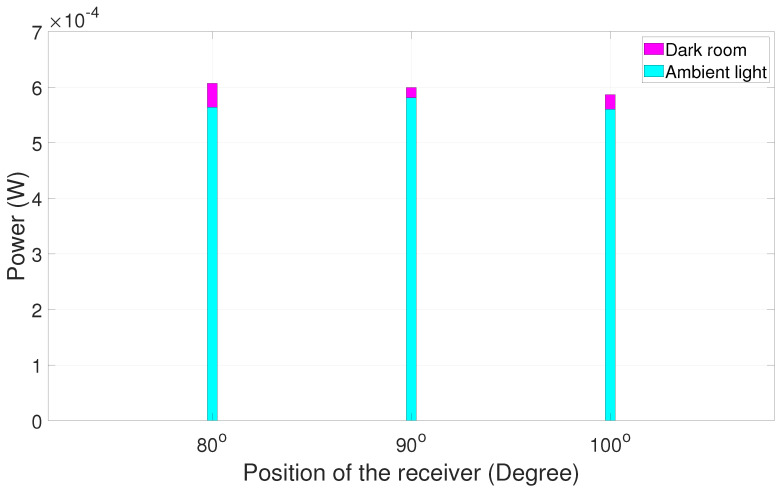
Dark room vs. ambient light power chart trial 4.

It is evident that, when the receiver is positioned at the large angular distance from the alignment position, the signal has maximum power at 90°, i.e., at the line-of-sight position. It is easy for the receiver to choose 90° because of the huge power dip on either side, like 30°, 40°, 150°, and 140° as shown in Figure 20. However, when the angular distance is reduced between respective positions (Figure 21), the power on either side is also highly comparable to 90°. Several experimental trials show that a minor synchronization issue leads to loss of data since the receiver is not pointing at a particular direction. Nevertheless, the received signal has good quality for processing. A similar experiment was carried out in a dark room and then in ambient light in order to study the comparison between the power values at two different scenarios. The results are summarized in Figure 22, Figure 23, Figure 24 and Figure 25.

The graphical depiction of the result explains that, when the experiment is carried out in the dark room, the power calculated is higher compared to the same experiment carried out in ambient light.

## 9. Discussion

This study is based on the adaptive alignment system’s performance under LOS and NLOS conditions. It analyzes the BER and power characteristics for manually changing the receiver position and extends to adaptive position change for LOS conditions using a servo motor. In both cases, experiments comprised several trials with varying angular displacement to explore the ability of this innovative UVLC approach to maintain reliable communication in the face of synchronization challenges. The result for the manual angular displacement for LOS shows that there is a considerable power dip when the receiver points at an angle away from the alignment position. The result is consistent with prior expectations that LOS communication typically provides the strongest signal, increasing the BER for the position not aligned with the transmitter. For NLOS communication, the adaptive system’s performance depends on the receiver’s FOV and the transmitter’s beam angle. The results highlight that even a slight misalignment can substantially degrade the system’s performance.

After retrieving the results for the BER and power analysis for LOS and NLOS communication, trials on the adaptive alignment system were performed for different angular displacements at the receiving end. However, challenges arose due to synchronization issues and the repetitive reconfiguration of the USRPs when switching between transmitter and receiver modes, which require careful time calibration at both ends for accurate transmission and data fetching, thus adding delays and instability, leading to minor data loss, particularly when the receiver was not positioned accurately in the optimal direction during the time of data reception. The proposed approach produced reasonable results, indicating that the designed model could achieve more reliable performance with further research.

Furthermore, opting for PPM is an effective approach for preserving signal accuracy without sacrificing energy efficiency. This modulation technique helps to improve the reliability of the UVLC system since it has better noise performance, but choosing the right PPM level is crucial since a high modulation level also increases the BER.

### Analysis

After several experimental trials, it was deduced that adaptive communication is sensitive to external environmental factors like background light. When external light falls on either side of the respective aligned position, the power received changes. This indicates that the power measurement results shown in Figure 17, Figure 20, and Figure 21 are actually the power of the received signal plus the background light. This explains why the power values are not identical at angles symmetrically offset by the same amount from 90 degrees. Performing the experiment in a dark room would give more exact results on received power. However, the signal can still be received with minor loss. Overall, this paper’s findings suggest that transmitting an optical signal via PPM using Li-Fi technology and an SDR gives reasonable preliminary results that need further refinement, particularly regarding synchronization accuracy. Better synchronization algorithms could mitigate the challenges faced during data transmission and reception.

## 10. Conclusions

UVLC is emerging as a promising solution for underwater communication. Accurate data transmission depends on the precise positioning of both the receiver and transmitter. By carefully selecting the optimal PPM level and angular alignment to minimize signal degradation, UVLC systems have the potential to outperform existing underwater communication technologies. NLOS communication is also achievable with UVLC. Real-time experiments suggest that the BER is zero when the alignment is perfect for the adaptive system, but the BER approaches 0.3 with 80-degree misalignment. Nevertheless, the performance could be further improved with a better receiver with a wide field of view to accommodate the transmitted beam. Additionally, the experimental trials using Li-Fi, an SDR, and a positional servo motor demonstrate that the adaptive system design is practical and convenient. Future improvements can be realized by implementing robust algorithms that enhance synchronization and overall system performance. In conclusion, the adaptive alignment system demonstrates strong potential for real-time UVLC applications, but challenges related to synchronization and power differentiation at small angular displacements must be addressed to enhance its performance in both LOS and NLOS conditions.

## Figures and Tables

**Table 1:** Overview of relevant studies and research gap.

Study	Focus	Research Gap Addressed
Tavares et al. [8]	Angle diversity and rate-adaptive technique for visible wireless communication.	Angle diversity technique at the receiver end for UVLC.
Kaymak et al. [9]	Adaptive beam steering technique free-space optical systems in high-speed trains.	1. Adaptive beam by changing receiver’s position in UVLC. 2. Servo motor and Arduino integration.
Simpson [10]	1. Smart transceiver design. 2. On–off keying code division multiple access (OOK-CDMA) modulation.	1. Adaptive UVLC system is implemented using SDR technology. 2. The receiver calculates the power and adapts to the direction of maximum beam intensity. 3. PPM modulation scheme.
Vali et al. [11]	1. Misalignment addressed. 2. Adapting beam divergence angle. 3. Monte-Carlo-based simulation.	1. Real- time UVLC system implemented by adapting the beam divergence with changing receiver’s position. 2.Li-Fi- and SDR-based UVLC system model is introduced.
Schirripa et al. [7]	Comparing various underwater technologies and the modulation schemes used.	Using PPM as modulation scheme for adaptive UVLC network.
Sun et al. [12]	1. Addressing the misalignment in UWOC. 2. Considering NLOS UWOC. 3. OOK modulation scheme used.	1. SDR-based technology. 2. Li-Fi receiver. 3. PPM modulation.
Xu et al. [13]	1. Diffraction beam shaping technique for UWOC. 2. Adjusting transmitter distance dynamically.	1. SDR-based. 2. Adjusting receiver position using servo motor and Arduino.
Salman et al. [14]	1. Relay-based approach. 2. Expanding the optical receiver’s reception area.	1. SDR-based approach. 2. Fixed reception area but adaptive receiver position, thus making the system more cost-efficient.
Palitharathna et al. [15]	1. Relay-based implementation. 2. OOK modulation.	1. SDR-based. 2. PPM modulation.
Rong et al. [16]	Simulation-based multi-hop UWOC network.	Real-time hardware setup with dynamic receiver position.
Chen and Rong [17]	1. SDR-based. 2. OFDM modulation.	1. SDR-based. 2. PPM modulation. 3. Addressing the misalignment using servo motor assembly.
Rehman et al. [4]	Designing a smart transceiver by changing the receiver position manually to find the BER and power curves.	Adaptive SDR-based transceiver model proposed for robust and efficient communication.

## Data Availability

Data is available upon request.
